# Performance, Carcass Traits, Pork Quality and Expression of Genes Related to Intramuscular Fat Metabolism of Two Diverse Genetic Lines of Pigs

**DOI:** 10.3390/foods11152280

**Published:** 2022-07-30

**Authors:** Marcos Henrique Soares, Gustavo de Amorim Rodrigues, Dante Teixeira Valente Júnior, Caroline Brito da Silva, Thaís Correia Costa, Marcio de Souza Duarte, Alysson Saraiva

**Affiliations:** 1 Department of Animal Sciences, Universidade Federal de Viçosa, Viçosa 36570-900, Brazil; marcos.henrique@ufv.br (M.H.S.); gustavo.a.rodrigues@ufv.br (G.d.A.R.); dante.junior@ufv.br (D.T.V.J.); caroline.brito@ufv.br (C.B.d.S.); thais.correia@ufv.br (T.C.C.); alysson.saraiva@ufv.br (A.S.); 2 Muscle Biology and Nutrigenomics Laboratory, Department of Animal Sciences, Universidade Federal de Viçosa, Viçosa 36570-900, Brazil; 3 Department of Animal Biosciences, University of Guelph, Guelph, ON N1G-2W1, Canada

**Keywords:** genetic line, growth performance, lipid metabolism, meat quality

## Abstract

We aimed to evaluate the performance, carcass and pork quality traits, as well as the mRNA expression of genes related to intramuscular fat deposition in female pigs from different genetic lines. A total of eighteen female pigs (Large White × Landrace × Duroc × Pietrain) × (Large White × Landrace) (Hybrid) averaging 88.96 ± 3.44 kg in body weight and twelve female pigs (Duroc) × (Large White × Landrace) (Duroc) averaging 85.63 ± 1.55 kg in body weight were assigned to a completely randomized design experimental trial that lasted 45 days. Pigs from both genetic lines received the same diet, which was initially adjusted for their nutritional requirements from 0 to 17 days of age and subsequently adjusted for nutritional requirements from 17 to 45 days of age. The performance of pigs did not differ among groups (*p* > 0.05). Duroc pigs showed a lower backfat thickness (*p* < 0.03) and greater intramuscular fat content (*p* < 0.1). A greater mRNA expression of the peroxisome proliferator-activated receptor gamma gene (PPARγ, *p* = 0.008) and fatty acid protein translocase/cluster differentiation (FAT/CD36, *p* = 0.002) was observed in the *Longissimus dorsi* muscle of Duroc pigs. Similarly, a greater expression of PPARγ (*p* = 0.009) and FAT/CD36 (*p* = 0.02) was observed in the *Soleus* muscle of Duroc pigs. Overall, we observed that despite the lack of differences in performance between the genetic groups, Duroc pigs had greater intramuscular fat content than hybrid pigs. The increased intramuscular fat content was associated with an increase in the mRNA expression of key transcriptional factors and genes encoding enzymes involved in adipogenesis and lipogenesis in glycolytic and oxidative skeletal muscle tissues.

## 1. Introduction

Over decades, the genetic improvement of pigs has focused on lean tissue deposition and the decrease of backfat tissue deposition [1,2]. As a consequence of selecting for increased lean tissue, a progressive decrease in intramuscular fat development has occurred [3,4]. Because intramuscular fat content is highly correlated to juiciness, flavor and tenderness [5,6,7], meat quality-related traits began to be included in pig breeding programs, along with existing selection traits [8,9].

The choice of the paternal line can determine carcass traits and meat quality [10]. There are different strategies for the formation of paternal lines to improve pork quality without major negative impacts on animal performance, two of which are the use of selected pure breeds, such as Duroc, or the use of hybrids, such as double hybrid composed by Large White, Landrace, Duroc and Pietrain breeds [10].

The Duroc breed is commonly recognized for providing a more tender and marbled pork, being associated with high-quality meat [11,12] but with a lower performance than hybrid pigs. On the other hand, hybrids pigs have high growth rate and muscle deposition [13]. However, hybrids provide pork with low quality attributes (i.e., tenderness, marbling and juiciness) compared to Duroc. This imbalance of animal performance and pork quality attributes is a consequence of the negative correlation existing between high growth rate and meat quality [14,15]. However, limited information is presented regarding the comparison between the progeny of these genetic lines and differences in the intramuscular fat metabolism between these lineages.

The lipid metabolism is regulated by several transcription factors, such as the Peroxisome proliferator-activated receptors alpha and gamma (PPARα and PPARγ), CAAT/*enhancer binding protein α* (C/EBPα), Lipoprotein lipase (LPL), Translocase fatty acid/cluster differentiation (FAT/CD36), fatty acid binding protein 3 (*FABP3*), Adiponectin (ADIP), Hormone sensitive lipase (HSL) and Carnitine palmitoyl transferase-1(*CPT-1*). These factors determine the rates of adipocytes biosynthesis, lipolysis and lipogenesis, as well as the uptake, transport and oxidation of fatty acids (Cho et al., 2011; Li et al., 2017; Malgwi et al., 2022). Previous studies using transcriptomic and gene expression analysis in the muscle of pigs with different genotypes observed that the genes attributed to lipolysis and lipogenesis were differentially expressed, which may influence the final fat deposition (Ayuso et al., 2016; Óvilo et al., 2014). Therefore, we aimed to evaluate performance, carcass traits, pork quality and the expression of genes related to lipid metabolism in glycolytic and oxidative muscle tissues of two divergent genetic lines of pigs.

## 2. Materials and Methods

This study was approved by the Brazilian Ethics Committee on Animal Use (CEUAP/UFV—protocol no. 013/2018), according to the ethical principles of animal experimentation established by the National Council of Animal Experimentation Control (CONCEA).

### 2.1. Animals, Experimental Design and Diets

Eighteen female pigs (Large White × Landrace × Duroc × Pietrain) × (Large White × Landrace) (HYB), with an initial body weight of 88.96 ± 3.44 kg, and twelve female pigs Duroc × (Large White × Landrace) (DUR), with an initial body weight of 85.63 ± 1.55 kg, were used. All animals were originally from a commercial pig-breeding farm and bought at the same time for the research trial.

Pigs were assigned into a completely randomized design where the pen represented the experimental unit, with two pigs per pen. As such, the HYB group had n = 9 (18 animals at total) and the DUR group had n = 6 (12 animals at total). The experiment lasted 45 days and the animals had free access to feed and water throughout the experimental period. The non-slated pen where the pigs were housed had dimensions of 2.30 × 2.10 m, and each one contained a dry feeder and a nipple drinker. During the trial, average temperature and humidity inside the barn measured by data logger were 22.04 ± 2.66 °C and 79.47 ± 0.1%, respectively.

The experimental period was divided into two phases: a phase 1 (0 to 17 days) and phase 2 (18 to 45 days) for the better adjustment of the nutritional requirements of the animals. The corn and soybean meal-based diets used in phase 1 and phase 2 (Table 1 and Table 2) were formulated to meet the requirements of the pigs, according to Rostagno et al. [16], except for standardized ileal digestible (SID) lysine in phase 2, which was supplemented with ractopamine. Considering that the addition of ractopamine increases the protein requirement, the SID lysine of the diets was increased by 20% [17,18]. Essential amino acids were included to maintain the ratios with SID lysine as recommended by Rostagno et al. [16]. All animals were fed the same diet in both phases.

Animals were fed three times per day and the total amount of feed provided during the trial was registered. Every morning the leftovers were manually collected and weighed to determine average daily feed intake (ADFI) at the end of the trial. At the end of the experimental period, piglets were individually weighed to determine final body weight (BW), average daily gain (ADG) and feed:gain ratio (F:G).

### 2.2. Blood Sampling and Analysis

On the last day of the trial period pigs were submitted to a 14 h fasting prior blood sampling. Blood was collected via venipuncture in the orbital sinus using needles (40 × 1.6 mm) and placed in 10 mL uncoated vacuum tubes. Samples were immediately sent to the Viçosa Clinical Laboratory (Viçosa, MG, Brazil) to measure the concentration of serum urea nitrogen (SUN), triglycerides (TG) and total cholesterol (CHO) (Ureal Cobas c 311, Roche Diagnostics GmbH, Basel, Switzerland).

### 2.3. Slaughter Procedures and Tissue Sampling

One pig from each pen with body weight closest to 140 kg, was stunned by electronarcosis and exsanguinated.

Immediately after exsanguination, samples of *Longissimus dorsi* (LD) and *Soleus* muscle (SM) were collected, placed in cryogenic tubes, snap-frozen in liquid nitrogen and stored at −80 °C.

All carcasses were weighed to measure hot carcass weight (HCW) and carcass yield (CY). The carcasses were divided longitudinally and refrigerated at 5 °C for 24 h. All measurements and sampling were performed on the left half of each carcass. The pH was measured at 15 min, 45 min, 1 h, 3 h, 6 h, 9 h, 12 h and 24 h after slaughter at the level of the last lumbar vertebra of the *Longissimus dorsi* (LD), using an appropriate pH meter (Testo SE & Co., Lenzkirch, FR, Germany).

After 24 h of cooling, 20 cm loin samples were collected of each left half of the carcasses, between the tenth rib and the first lumbar vertebra for meat quality traits evaluation. The LD samples were stored at −20 °C for 24 h and then sectioned into five 2.54 cm thick chops, which were vacuum packed and stored at −20 °C for further analysis [19].

### 2.4. Carcass Traits

At the 10th rib, the left half of each carcass was sectioned and the backfat thickness (6 cm away from the midline) was measured using a digital caliper. To determine loin muscle area (LMA), the muscular surface of the LD between the 10th and 11th rib was covered with a polyethylene sheet and contoured using a permanent fine-tipped marker. The sheets were digitally scanned and colored. Colored areas within the contour were measured using an image analysis software (ImageJ version 1.49 t, National Institutes of Health, Bethesda, Rockville, MD, USA). HCW and CY were obtained soon after slaughter, as described above.

### 2.5. Pork Quality

Thawing loss (THL), cooking loss (CL) and total (TL) determination were performed according to Soares et al. [20].

After weighing, cooked chops were used to Warner–Bratzler shear force (WBSF) determination as proposed by American Meat Science Association [21] with minor modifications as described by Soares et al. [20].

The collected loin of left half of each carcass were allowed to bloom for 30 min for color evaluation. Then, meat color was measured using a spectrophotometer (Hunter MiniScan EZ, 4500 L; Hunter Associates Laboratory, Inc., Reston, VA, USA), calibrated against a white and a black tile. The mean L * (lightness), a * (redness) and b * (yellowness) values of each chop were determined as the average from 3 readings on 3 different points of chop surface, using illuminant D65, a 31.8 mm port size and a 10° standard observer [22,23].

To determine intramuscular fat (IMF) the protocol described by Soares et al. [20] was followed without modifications.

### 2.6. Gene Expression Analysis

For gene expression analysis the total RNA extraction was performed by using Trizol^®^ (ThermoFisher Scientific, Beverly, MA, USA) following the manufacturer protocol. After extraction, total RNA concentration was determined by using a NanoDropTM Lite spectrophotometer (ThermoFisher Scientific, Beverly, MA, USA). The integrity of total RNA was checked by using 1% agarose gel. After quality check, total RNA was reverse-transcribed into cDNA by using a Go Script Reverse Transcription kit (Promega, Madison, WI, USA) according to the manufacturer protocol.

Primers used for RT-qPCR of the target and housekeeping genes are described in Table 3. RT-qPCR reactions were performed in QuantStudio^®^ 3 (Applied Biosystems, Thermo Fisher Scientific).

The threshold cycle (Ct) values obtained were normalized (ΔCt) based on the Ct value of glyceraldehyde-3-phosphate dehydrogenase. Gene of interest relative expression was calculated by 2^−ΔΔCt^ [24].

### 2.7. Statistical Analysis

For performance (FBW, ADFI, ADG and F:G) data analysis, the pen was considered the experimental unit, the initial body weight was used as a covariate. One pig per pen was considered the experimental unit for the other analysis. For the analysis of carcass traits (HCW, CY, LMA and BF) FBW was used as covariate.

Statistical analysis was performed using the R software (version 3.4.4, 2017, R Core Team 2018, R Foundation for Statistical Computing, Vienna, Austria, https://www.r-project.org/, accessed on 12 June 2021). Data were subjected to analysis of variance (ANOVA). Significant results were considered at *p* ≤ 0.05.

## 3. Results

### 3.1. Performance and Serological Analysis

There was no effect (*p* > 0.05) on the final body weight (FBW), average daily feed intake (ADFI) and feed conversion ratio (F:G) between Duroc and Hybrid pigs (Table 4).

Indeed, the levels of total cholesterol (CHO), triglycerides (TG) and serum urea nitrogen (SUN) did not differ (*p* > 0.05) between Duroc and Hybrid pigs (Table 5).

### 3.2. Carcass Traits

There was no difference (*p* > 0.05) between hot carcass weight (HCW), carcass yield (CY) and loin muscle area (LMA) between Duroc and Hybrid pigs. However, Duroc pigs had lower (*p* = 0.03) back fat thickness (BF) compared to Hybrid animals (Table 6).

### 3.3. Carcass pH

There was no difference (*p* > 0.05) in carcass pH values between Duroc and Hybrid pigs (Table 7).

### 3.4. Pork Quality

The pork from Duroc pigs had greater values of (*p* = 0.04) L* (brightness) and the lowest values (*p* < 0.03) a* (redness) compared to Hybrid pigs. The b* (yellowness) value did not differ (*p* > 0.05) between Duroc and Hybrid pigs (Table 8).

A similar value of Warner–Bratzler shear force (WBSF) was observed (*p* > 0.05) between Duroc and Hybrid pigs. On the other hand, Duroc pigs had a higher (*p* < 0.001) percentage of IMF compared to Hybrid pigs.

Thawing losses (THL), cooking losses (CL) and total losses (TL) did not differ between Duroc and Hybrid pigs (Table 8).

### 3.5. mRNA Expression in Longissimus Dorsi Muscle

Greater mRNA abundance of *PPARγ* (*p* = 0.008), *FAT/CD 36* (*p* = 0.002) and the HSL (*p* = 0.01) and ADIP (*p* = 0.002) was observed in DUR pigs (Table 9).

### 3.6. mRNA Expression Soleus Muscle

Greater mRNA abundance of *PPARγ* (*p* = 0.009) and *FAT/CD 36* (*p* = 0.02) was observed in DUR pigs (Table 10).

## 4. Discussion

The adequate behavior of pH decline of the carcass is relevant for the transformation of muscle into quality meat, directly impacting on sanitary aspects, such as shelf life and microbiological proliferation, as well as water retention capacity and the perception of meat color by consumers [25,26]. In this study, although the genetic groups may have different muscle deposition potentials and, thus, diverse concentrations of glycogen stored in the muscle tissue, no difference was observed in the declining of carcass pH between both groups, indicating that the use of DUR or HYB pigs does not affect the postmortem pH declining over time, which is in agreement with Morcuend et al. (2007) and Alonso et al. (2015).

Although no effect on pH was observed, meat from DUR pigs had higher L* and lower a* values compared to pigs from HYB group, indicating greater luminosity and lower red intensity. Although studies have shown no changes in color parameters (Latorre et al., 2003; Alonso et al., 2009, 2015) similar to the present study, McGloughlin et al. (1998) observed that the meat of Duroc was slightly paler than the meat of the Large White. The meat quality parameters may be sustained, at least partially, by the variation in fiber type characteristics [27]. For instance, the increase in the proportion of fast glycolytic 2b fibers is correlated with the increase in meat luminosity values [28]. Furthermore, glycolytic fibers have lower concentration of myoglobins, resulting in a less intense red color than muscles with high concentration of myoglobins [29]. Moreover, it was reported a significant positive and negative correlation between the color parameter a* (redness) and the type 2a and 2b muscle fibers in LD muscle of Duroc pigs, respectively, consistently with the previous knowledge that the isoform MyHC 2b is the most glycolytic and least efficient in terms of oxygen exchange compared to the other isoforms [24]. This fact may be an indicative that the incorporation of pure Duroc breeds in a population may contribute to the enhancement of the glycolytic metabolism in the muscle of the upcoming generations.

The quality of meat is perceived by consumers for its visual and sensory traits. Among sensory traits, flavor, juiciness and tenderness are essential to determine meat quality, and a visual trait that is directly related to sensory ones is the intramuscular fat content. Thus, intramuscular fat is very important for the quality of meat because it mainly intensifies juiciness and flavor [11,30]. However, the greatest deposition of intramuscular fat occurs in the later stages of the growth process. Hence, the achievement of this fat depots without the enhancement in other fat depots is challenging. The potential of intramuscular fat deposition in Duroc pigs compared to other pig lineages has been previously reported by other studies [11,12,31]. In the current study, the pork chops from DUR pigs had a higher concentration of intramuscular fat and lower values of backfat thickness. Evaluating these characteristics in Duroc pigs, Zhang et al. [32] were able to identify specific loci and genes through the single-step genome-wide association (ssGWAS) approaches that affect the intramuscular fat without changing the backfat content, providing potential markers for breeding programs aiming to improve pork quality in term of fat deposition.

Another important sensory trait for meat palatability is tenderness [33], measured by the Warner–Bratzler shear force (WBSF). In the present study, WBSF of meat was similar between HYB and DUR pigs; however, there was a trend towards a reduction in WBSF in DUR pigs. This trend may be due to the higher concentration of intramuscular fat observed in DUR pigs, since there is a negative correlation between intramuscular fat content and meat tenderness [34]. Previous studies (Morlein et al., 2007, Jelenikova et al., 2008; Alonso et al., 2015) reported that an increasing Duroc in crossbreeding reduced the WBSF, which is in agreement with our results. Although the greater deposition of intramuscular fat was evidenced in DUR pigs in the current study and others [11,35,36] compared to a variety of pig lineages, little is known about the differences in the molecular mechanisms associated to intramuscular fat deposition in DUR and HYB pigs. Evaluating the markers related to adipogenesis, lipolysis and oxidative processes, we observed a greater abundance of the genes PPARγ, FAT/CD36, HSL and ADIP in the LD of DUR pigs. Similar results were obtained by Zhao et al. (2020) through transcriptome analysis, indicating potential genes that affect IMF content in purebred Duroc pigs. PPARγ is crucial for the processes of adipogenesis and lipogenesis, while trans-membrane fatty acid transport protein encoded by FAT/CD36 is regulated by PPARγ [37] and plays a role in lipid accumulation. Thus, its greater abundance in muscle tissue may be associated with the increase in the number and hypertrophy of adipocytes, resulting in the accumulation of intramuscular fat [38,39].

When necessary, the energy stored as intramuscular fat in muscle tissue [40] is mobilized trough the activation of the lipolytic enzymes, such as HSL, and the lipolysis process is initiated. Thus, the increased expression of HSL and ADIP may be related to the greater use of intramuscular fat as a source of energy in muscle tissue.

The CPT-1 enzyme catalyzes the formation of the Acyl-carnitine complex, which acts on the β-oxidation of long-chain fatty acids. Thus, CPT-1 is described as a key regulator of mitochondrial β-oxidation [41,42]. In the present study, there was a trend (*p* = 0.06) of increased expression of the CPT-1 enzyme in DUR pigs, indicating that in these animals, despite the accumulation of intramuscular fat, there was also a higher expression of enzymes related to β-oxidation, which suggests that these pigs possibly use a greater proportion of intramuscular fat as an energy source compared to HYB pigs.

The increase of ADIP concentration in skeletal muscle tissue is often associated with increased β-oxidation [43]. However, interestingly, Wang et al. [44] demonstrated a positive correlation between ADIP mRNA abundance and intramuscular fat content in Chinese Laiwu pigs, which is consistent with what was observed in the present study, suggesting that in DUR pigs the greater expression of ADIP in LD is due to the increase in intramuscular fat, since ADIP can be produced by adipocytes, including intramuscularly.

In SM, characterized by its oxidative properties, the gene expression of enzymes and transcription factors related to lipogenesis followed a similar behavior observed in LD. Duroc pigs had a greater abundance of PPARγ and FAT/CD 36 mRNA in the SM, indicating a greater lipogenic potential compared to HYB group, which is in agreement with the grater IMF observed.

## 5. Conclusions

Pigs from both genetic groups showed a similar performance. However, DUR pigs had lower backfat thickness and greater intramuscular fat content. The greater intramuscular fat content observed was associated with an increase in the mRNA expression of key transcriptional factors and genes encoding enzymes involved in adipogenesis and lipogenesis in glycolytic and oxidative skeletal muscle tissues. The genetic mechanisms underlying the association of the phenotypical traits and mRNA expression of the intramuscular fat metabolism in the genetic groups evaluated in this trial remains to be explored.

## Figures and Tables

**Table 1:** Diets composition.

Ingredient, %	Phase 1	Phase 2
Corn	72.325	72.325
Soybean meal	20.000	20.000
Soybean oil	3.550	3.550
Dicalcium phosphate	0.950	0.950
Limestone	0.830	0.830
Inert clay filler	1.200	0.595
Salt	0.355	0.355
L-lysine HCl, 98.5%	0.230	0.435
DL—methionine, 99.0%	0.050	0.145
L—threonine, 98.5%	0.035	0.155
L—tryptophan, 98.0%	0.015	0.045
L—valine, 96.5%	---	0.055
Mineral premix ^1^	0.200	0.200
Vitamin premix ^2^	0.200	0.200
Antibiotic ^3^	0.050	0.050
Ractopamine ^4^	---	0.100
Antioxidant ^5^	0.010	0.010

^1^ Content per kg: Fe as Fe_2_(SO_4_)_3_ (15.0 g), Cu as CuSO_4_ (40.0 g), I as Ca(IO_3_)_2_ (350.0 mg), Zn as ZnO (25.0 g), Mn as MnSO₄ (13.0 g). ^2^ Content per kg: folate (125.00 mg), pantothenic acid (4000 mg), biotin (12.50 mg), niacin (825.00 mg), Se (75.00 mg), vit B6 (250.00 mg), vit B2 (1350.00 mg), vit B1 (250.00 mg), vit A (2,100,000 U.I.), vit B 12 (6000.00 mcg), vit D3 (350,000 U.I.), vit E (5.000 U.I.), vit K3 (850.00 mg). ^3^ Provided per kg of diet: 3250 g tylosin (Tylan G250, Elanco, São Paulo, SP, Brasil). ^4^ 20 g ractopamine/kg (Ractomax, Sauvet, Campinas, SP, Brasil). ^5^ 865 g butylated hydroxytoluene/kg, 15 g ethoxyquin/kg and 7 g butylated hydroxy anisole/kg (Banox 100, Alltech Inc., Nicholasville, KY, USA).

**Table 2:** Diet calculated nutritional composition ^1^.

Item	Phase 1	Phase 2
ME, kcal/kg	3.350	3.350
Crude protein, %	15.00	15.44
SID Lys, %	0.811	0.973
SID Met + Cys, %	0.487	0.584
SID Thr, %	0.527	0.632
SID Trp, %	0.162	0.195
SID Val, %	0.623	0.671
SID Iso, %	0.546	0.546
Sodium, %	0.158	0.158
Calcium, %	0.545	0.545
Available phosphorus, %	0.269	0.269

^1^ Values calculated according to Rostagno et al. [16]. SID = standardized ileal digestible.

**Table 3:** Oligonucleotides used on gene expression analysis.

Gene	GenBank No.	Sequence	Size, bp
GAPDH	AF017079	F:5′-CCTTCCGTGTCCCTACTGC-3′ R:5′-CATCAAAGGTAGAAGAGTGAGTGTC-3′	195
CPT1	NM_001007191.1	F:5′-GGACGAGGAGTCTCACCACTATGAC-3′ R:5′-TCTTGAACGCGATGAGGGTGA-3′	128
PPARγ	NM_214379	F:5′-GTGGAGACCGCCCAGGTTTG-3′ R:5′-GGGAGGACTCTGGGTGGTTCA-3′	108
PPARα	NM_001044526.1	F:5′-GGCTTACGGCAATGGCTTCA-3′ R:5′-CGGTCTCCGCACCAAATGA-3′	168
C/EBPα	AF103944	F:5′-CGTGGAGACTCAACAGAAGG-3′ R: 5′-GCAGCGTGTCCAGTTCGCGG-3′	95
LPL	NM_214286.1	F:5′-CCCTATACAAGAGGGAACCGGAT-3′ R:5′-CCGCCATCCAGTCGATAAACGT-3′	138
FAT/CD36	NM_001044622.1	F:5′-CTGGTGCTGTCATTGGAGCAGT-3′ R:5′-CTGTCTGTAAACTTCCGTGCCTGTT-3′	160
FABP3	NM_001099931.1	F:5′-CCAACATGACCAAGCCTACCACA-3′ R:5′-ACAAGTTTGCCTCCATCCAGTGT-3′	176
ADIP	AY135647	F:5′-GGAGATACAGGTCTTACTGGTCCTA-3′ R:5′-CAGGAATGTTGCAGTGGAATTTGCCA-3′	262
AdipoR1	NM_001007193	F:5′-GACCATGCTCAGACCAAATATGTATTTCA-3′ R:5′-AACGAGTAATAGAGCCAGGGGACGAAGCT-3′	222
AdipoR2	NM_001007192	F:5′-CAAACATCTCCTTTGTGGCCC-3′ R:5′-CAAGTAGATGAAGCAAGGTTGCGGGTTA-3′	242
HSL	AJ000482	F:5′-GCT CCC ATC GTCAAG AAT C-3′ R:5′-TAA AGC GAA TGCGGT CC-3′	112

Glyceraldehyde-3-phosphate dehydrogenase (*GAPDH*); Carnitine palmitoyl transferase-1(*CPT-1*); Peroxisome proliferator activated gamma receptor (PPARγ); Peroxisome proliferator activated receptor alpha (PPARα); CAAT/*enhancer binding protein α* (C/EBPα); Lipoprotein lipase (LPL); Translocase fatty acid/cluster differentiation (FAT/CD36); fatty acid binding protein 3 (*FABP3*); Adiponectin (ADIP); Adiponectin receptor 1 (AdipR1); Adiponectin receptor 2 (AdipR2); Hormone sensitive lipase (HSL).

**Table 4:** Performance of pigs of different genetic lines.

Item ^1^	HYB	DUR	SEM ^3^	*p*-Value
IBW, kg ^2^	88.90	85.63	0.73	0.06
FBW, kg	144.68	143.65	1.54	0.39
ADFI, kg	2.860	3.020	0.05	0.26
ADG, kg	1.238	1.289	0.03	0.38
F:G	2.37	2.37	0.03	0.88

^1^ IBW = initial body weight, FBW = final body weight, ADFI = average daily feed intake, ADG = average daily gain, F:G = feed conversion ratio. ^2^ IBW was used as a covariate. ^3^ Standard error of the mean. HYB = Hybrid; DUR = Duroc.

**Table 5:** Concentration of serum metabolites of pigs of different genetic lines.

Item ^1^	HYB	DUR	SEM ^2^	*p*-Value
CHO, mg/dL	91.00	88.00	2.06	0.50
TG, mg/dL	31.00	27.17	2.15	0.41
SUN, mg/dL	22.44	23.83	1.19	0.59

^1^ CHO = total cholesterol; TG = triglycerides; SUN = serum urea nitrogen. ^2^ Standard error of the mean. HYB = Hybrid; DUR = Duroc.

**Table 6:** Carcass traits of pigs of different genetic lines.

Item ^1^	HYB	DUR	SEM ^3^	*p*-Value
HCW ^2^, kg	120.52	116.87	1.05	0.12
CY, %	83.74	82.74	0.38	0.24
BF, mm	13.86	11.49	0.68	0.03
LMA, cm^2^	73.33	68.89	1.46	0.19

^1^ HCW = hot carcass weight; CY = carcass yield; LMA = loin muscle area; BF = backfat thickness. ^2^ FBW was used as covariate for hot carcass traits. ^3^ Standard error of the mean. HYB = Hybrid; DUR = Duroc.

**Table 7:** Carcass pH of pigs of different genetic lines.

Item	HYB	DUR	SEM ^1^	*p*-Value
pH				
15 min	6.593	6.720	0.05	0.19
45 h	6.353	6.513	0.04	0.09
1 h	6.227	6.487	0.08	0.16
3 h	5.848	6.048	0.08	0.28
6 h	5.660	5.798	0.06	0.30
9 h	5.564	5.532	0.03	0.65
12 h	5.503	5.486	0.02	0.66
24 h	5.461	5.471	0.01	0.69

^1^ Standard error of the mean. HYB = Hybrid; DUR = Duroc.

**Table 8:** Pork quality parameters evaluated on LD of pigs of different genetic lines.

Item ^1^	HYB	DUR	SEM ^2^	*p*-Value
THL, %	7.399	6.360	0.43	0.28
CL, %	15.460	15.100	0.52	0.77
TL, %	22.285	20.489	0.47	0.09
WBSF, kgf	4.340	3.926	1.19	0.07
L*	54.001	56.808	0.59	0.04
A*	6.836	5.610	0.25	0.03
B*	13.265	13.532	0.19	0.51
IMF, %	1.446	1.933	0.05	<0.001

^1^ THL = thaw water losses; CL = cooking water losses; TL = sum of water losses; WSBF = warner-Bratzler shear force; IMF = intramuscular fat content. ^2^ Standard error of the mean. HYB = Hybrid; DUR = Duroc.

**Table 9:** mRNA expression in the LD muscle of pigs of different genetic lines.

Item	HYB	DUR	SEM ^1^	*p*-Value
PPARγ	1.236	2.034	0.12	0.008
CEBPα	5.489	5.375	0.52	0.92
FAT/CD 36	1.497	2.817	0.16	0.002
FABP3	1.405	1.198	0.09	0.27
LPL	1.507	2.444	0.26	0.11
HSL	3.471	5.782	0.37	0.01
PPARα	2.053	2.337	0.18	0.47
CPT-1	1.247	1.852	0.14	0.06
ADIP R1	2.336	1.758	0.31	0.39
ADIP R2	8.907	11.608	0.99	0.22
ADIP	1.224	3.475	0.28	0.002

^1^ Standard error of the mean. HYB = Hybrid; DUR = Duroc.

**Table 10:** mRNA expression in SM muscle of pigs of different genetic lines.

Item	HYB	DUR	SEM ^1^	*p*-Value
PPARγ	1.243	2.310	0.17	0.009
CEBPα	6.008	4.728	1.17	0.61
FAT/CD 36	1.941	2.936	0.18	0.02
FABP3	8.270	9.472	0.32	0.10
LPL	1.603	2.096	0.21	0.28
HSL	8.407	7.026	1.08	0.56
PPARα	13.448	9.345	1.01	0.08
CPT-1	2.768	2.394	0.43	0.69
ADIP R1	13.278	14.956	0.72	0.29
ADIP R2	5.213	7.930	1.18	0.30
ADIP	14.505	11.671	1.32	0.39

^1^ Standard error of the mean. HYB = Hybrid; DUR = Duroc.

## Data Availability

The data presented in this study are available on request from the corresponding author.
